# Bio-Fabrication of Silver Nanoparticles Using *Citrus aurantifolia* Fruit Peel Extract (CAFPE) and the Role of Plant Extract in the Synthesis

**DOI:** 10.3390/plants12081648

**Published:** 2023-04-14

**Authors:** Tijjani Mustapha, Nur Raihana Ithnin, Hidayatulfathi Othman, Zatul-’Iffah Abu Hasan, Norashiqin Misni

**Affiliations:** 1 Department of Medical Microbiology, Faculty of Medicine and Health Sciences, Universiti Putra Malaysia, Serdang 43400, Selangor Darul Ehsan, Malaysia; 2 Department of Biological Sciences, Faculty of Science, Yobe State University, Damaturu P.M.B 1144, Yobe State, Nigeria; 3 Department of Biomedical, Faculty of Health Sciences, Universiti Kebangsaan Malaysia, Bandar Baru Bangi, Bangi 43600, Selangor, Malaysia; 4 Faculty of Medicine and Health Sciences, Universiti Sains Islam Malaysia, Nilai 71800, Negeri Sembilan, Malaysia

**Keywords:** silver nanoparticles, *Citrus aurantifolia*, green synthesis, plant extract, metabolites

## Abstract

The green synthesis of silver nanoparticles has been proposed as an eco-friendly and cost-effective substitute for chemical and physical methods. The aim of this study was to synthesize and characterize silver nanoparticles using the peel extract of *Citrus aurantifolia* fruit, and to determine the possible phytochemical constituents’ presence in the plant extracts that might be responsible for the synthesis. *Citrus aurantifolia* fruit peel extraction was followed by phytochemical studies of secondary metabolites, FTIR analysis confirmation of functional groups, and GC–MS analysis. Silver nanoparticles were synthesized through bio-reduction of silver ions (Ag^+^) to silver nanoparticles using CAFPE and characterized using UV-Vis spectroscopy, HR–TEM, FESEM, EDX, XRD, DLS, and FTIR. The presence of plant secondary metabolites such as alkaloids, flavonoids, tannins, saponins, phenols, terpenoids, and steroids was detected. The FTIR analysis of the extract revealed the presence of functional groups like hydroxyl, carboxyl, carbonyl, amine, and phenyl, whereas the GC–MS analysis indicated presence of chemical compounds such as 1,2,4-Benzenetricarboxylic acid, Fumaric acid, nonyl pentadecyl, and 4-Methyl-2-trimethylsilyloxy-acetophenone, etc., with similar functional groups. The synthesized silver nanoparticle (AgNP) has displayed the characteristics of a surface plasmon resonance (SPR) band peak from 360–405 nm. High resolution transmission electron microscope (HR-TEM) and field emission scan electron microscope (FESEM) confirm polydisperse, spherical shaped, and smooth surface nanoparticles with an average size of 24.023 nm. Energy dispersive X-ray (EDX) analysis further revealed that silver is the most abundant element found in the micrograph of the nanoparticles, and FTIR analysis further confirmed the presence of different functional groups in the surface of the nanoparticle. The XRD analysis also confirmed that the nanoparticles synthesized are crystalline in nature. Based on the findings of this study, it is understood that the variety of natural compounds that are present in plant extracts of *Citrus aurantifolia* fruit peel can act as both reducing and stabilizing agents for the synthesis of silver nanoparticles. It is, therefore, concluded that *Citrus aurantifolia* peel extract can be potentially used for the large production of silver nanoparticles for several applications.

## 1. Introduction

In recent years, there has been growing interest in the synthesis of metallic nanoparticles such as zinc, silver, platinum, and gold nanoparticles due to their potential and possible applications in different fields such as nanomedicine, biomedicine, agriculture, and biosensors [1]. Silver nanoparticles are one of the most extensively studied nanomaterials due to their high stability and low chemical reactivity in comparison to other metals. They are widely used as anticancer, antibacterial, and larvicidal agents due to their promising and distinct properties [2,3]. However, they are commonly synthesized using two different methods: the physical and the chemical methods [4]. The physical method requires the use of sophisticated equipment, high energy, high pressure, and high temperature, which are costly. On the other hand, the synthesis of silver nanoparticles using chemical methods requires the use of toxic chemicals such as N, N-dimethyl formamide (DMF) and trisodium citrate. These chemicals are known to cause serious damage to the environment and to the living components of the environment [5]. Thus, there is a need to develop an eco-friendly method. 

Recently, a green technique has been suggested to substitute the nature-unfriendly chemical and physical methods. In the green synthesis technique or method, which is also known as the biological method, plants, fungi, and bacteria are used. Several studies have reported the green synthesis of silver nanoparticles using plant extracts from various parts of plants such as the peel, stem, leaf, root, and fruit [6,7,8,9,10,11,12]. Although the mechanism of silver nanoparticle synthesis is still not clear, it is generally speculated that the biomolecules contained in different plants can play an important role in the synthesis of silver nanoparticles by reducing metal ions and stabilizing the nanoparticles to the desired shapes and sizes [13,14]. The green synthesis method of silver nanoparticles offers several benefits, such as the elimination of harmful chemicals, the utilization of less energy, the generation of environmentally benign products and byproducts, and the simplicity of the process [15]. 

*Citrus aurantifolia* is a flowering plant in the family Rutacea. They are commonly called “bitter orange” or “key lime” [16]. In Malaysia, it is one of the most popular *Citrus* species, generally used in cuisine and traditional medicine. It has a spiny stem which is about 3–5 m tall. The *Citrus* plant has a round shape, and 5–9 cm-long leaves with 3–5 cm thickness [17]. It is widely distributed throughout South–East Asia, China, and India. It has been reported that the worldwide production of citrus fruit increased to 82 million tons in 2010, and about 70% of the world’s total marketable citrus fruits are grown in America, Brazil, Mediterranean countries. Of these, India is the world’s largest producer of different varieties of lime [18]. *Citrus aurantifolia* fruits are well recognized for their antioxidant properties due to their high content of secondary metabolites such as terpenoids, flavonoids, ascorbic acid, etc. The bioactive phytochemicals present in *Citrus aurantifolia* fruits are capable of functioning as antioxidants and it has been argued that the ability of a plant extract to reduce metal ions to nanoparticles is attributed to their antioxidant activities [19,20,21]. Furthermore, these bioactive compounds may play an important role in degenerative diseases caused by oxidative stress, such as cancer and Alzheimer’s disease [22].

In spite of several studies on silver nanoparticles, more comprehensive studies on the green synthesis of silver nanoparticles using plant extracts are required. To our knowledge, there has been no study on the plant-mediated synthesis of silver nanoparticles using *Citrus* *aurantifolia* fruit peel extract. Therefore, the novelty of the present study lies in identifying and describing the role of metabolites in the synthesis of silver nanoparticles. In the light of the above, the aim of this study was to synthesize and characterize silver nanoparticles using *Citrus aurantifolia* fruit peel extract (CAFPE) and to determine the possible phytochemical constituents’ presence in the plant extracts that might be responsible for the synthesis of the silver nanoparticles.

## 2. Results

### 2.1. Phytochemical Screening of Citrus aurantifolia Fruit Peel Extract

The phytochemical analysis of the *Citrus aurantifolia* fruit peel extract was conducted to determine the presence of bioactive compounds. The compounds detected are shown in Table 1. The results show the presence of seven (7) different secondary metabolites that include alkaloids, flavonoids, tannins, saponins, phenols, terpenoids, and steroids, whereas anthraquinones were absent. The extract contains important biomolecules that can effectively reduce silver ions into silver nanoparticles.

### 2.2. Gas Chromatography Mass Spectrometry (GC–MS) Analysis of the Plant Extract

**Figure 1:**
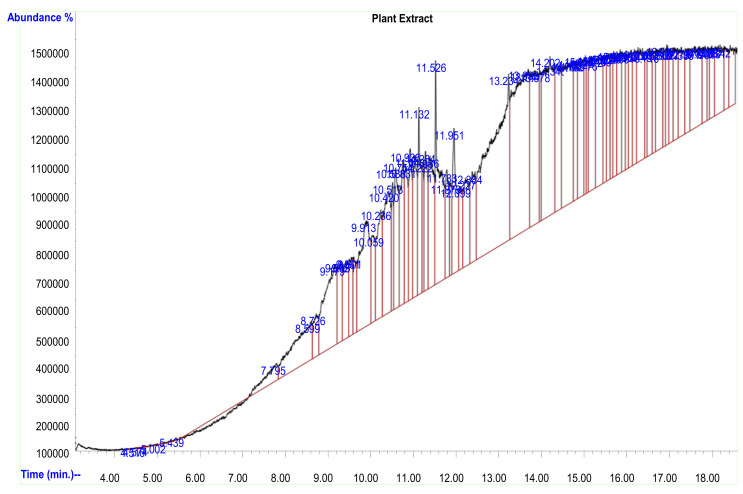
Spectrum of GC–MS analysis of *Citrus aurantifolia* fruit peel extract.

The gas chromatography mass spectrometry (GC–MS) analysis of the peel extract of the *Citrus aurantifolia* fruit was conducted to determine the bioactive chemical compounds present in the extract. The GC–MS spectrum of the extract with peaks and retention times is shown in Figure 1. The identification of the chemical compounds present in the plant extract was achieved by comparing their retention time and mass fragmentation pattern with those available in the NIST libraries. The analysis revealed the presence of seventy-three (73) chemical compounds representing 100% of the plant extract, with thirty-six (36) of them having a peak area percentage of <1% and thirty-seven (37) having a peak area of >1%. Ten (10) major chemical compounds were identified, with peak area percentage ranging from 2.10% to 8.16%. The chemical compounds include Tris (tert-butyldimethylsilyloxy) arsane (2.017%), Cyclotrisiloxane, hexamethyl- (2.10%), 7-Pentadecyne (2.16%), 2-Pyridinamine, N-(4,5-dihydro-5-m ethyl-2-thiazolyl)-3-methyl-7 (2.68%), Eicosane, 2-methyl- (2.63%), 4-Methyl-2-trimethylsilyloxy-acetophenome (3.55%), 1,2,4-Benzenetricarboxylic acid (4.42%), 1,1,1,3,5,5,5-Heptamethyltrisiloxane (6.48%), Fumaric acid, nonyl pentadecyl (2.81), and Cyclotrisiloxane, hexamethyl- (8.16%) (Table 2).

### 2.3. Fourier Transform Infrared Spectroscopy (FTIR) of Plant Extract

**Figure 2:**
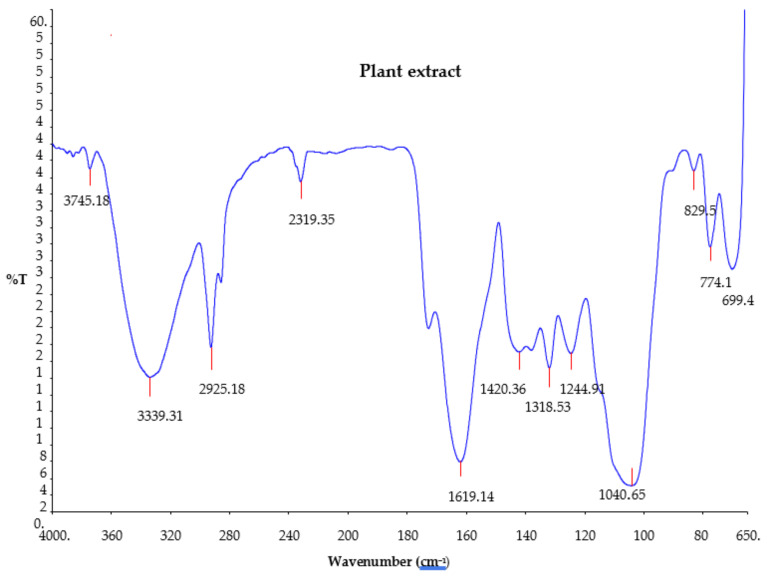
Fourier transform infrared spectrum of *Citrus aurantifolia* peel extract.

The Fourier transform infrared (FTIR) analysis of the *Citrus aurantifolia* plant extract was carried out to determine the functional groups’ presence in the plant extract. The results of the analysis are shown in Figure 2. The FTIR spectrum of the plant extract showed that 12 different absorption bands appeared in different regions, indicating the existence of several compounds in the plant extract. The resulting FTIR bands include 3745.1, 3339.3, 2925.1, 2319.3, 1619.1, 1420.3, 1318.5, 1244.9, 1040.6, 829.59, 774.15, and 699.5 cm^−1^. Broad peaks appearing at 3339.3 and 3745.1 cm^−1^ are due to COOH (carboxyl) and O-H (hydroxyl) stretching, which indicates the presence of alcohol and phenols. The bands at 2925.1, 2319.3, and 1420.3 cm^−1^ are due to C-H stretch (alkyne) [7,23,24]. Moreover, the peak at 1040.6 cm^−1^ is due to alcohol C-O stretch [7]. The band observed at 1619.1 cm^−1^ is due to C=O, which indicates the presence of carbonyl (esters) [25]. The absorption band peak observed at 1318.5 cm^−1^ is assigned to OH bending of phenols [26], whereas the bands at 829.59 cm^−1^, 774.15 cm^−1^, and 699.5 cm^−1^ are due to C=C of bend alkyne.

### 2.4. Biosynthesis of Silver Nanoparticles Using Citrus Aurantifolia Peel Extract

The biosynthesis of the silver nanoparticles occurs after adding the *Citrus aurantifolia* peel extract to the silver nitrate solution. The formation of the nanoparticles starts as the color of the solution changes from slightly yellow to dark brown. The color change was rapid, and it was observed virtually after 30 min of reaction time. This color change was linked to the reduction of silver ions (Ag^+^) to silver nanoparticles (AgNPs) by different bioactive compounds present in the plant extract that serve as reducing and stabilizing agents.

### 2.5. UV- Visible Spectroscopy 

**Figure 3:**
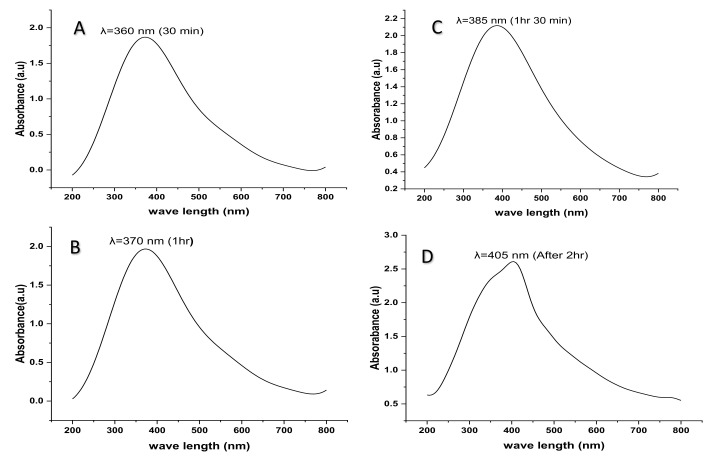
UV–visible absorption spectra of AgNPs synthesized.

The formation and synthesis of silver nanoparticles using *Citrus aurantifolia* fruit peel extracts were characterized using a UV-Visible spectrophotometer (Beckman Coulter DU730, Santiago, MI, USA) at different reaction time intervals of thirty minutes, one hour, one hour and thirty minutes, and two hours. The spectra were recorded in the wavelength of 200–800 nm. The synthesized silver nanoparticles have displayed the characteristics of a surface plasmon resonance (SPR) band peak between 360 nm and 405 nm. However, it has been observed that, after 30 min of the reaction, the absorption peak band of the colloidal silver nanoparticles appears at 360 nm, as shown in Figure 3. After 1 h, the maximum absorption peak band has shifted from 360 nm to a longer wavelength of 370 nm, 385 nm after 1 h and 30 min, and 405 nm after 2 h of reaction (Figure 3A–D).

### 2.6. High Resolution Transmission Electron Microscope (HR-TEM)

**Figure 4:**
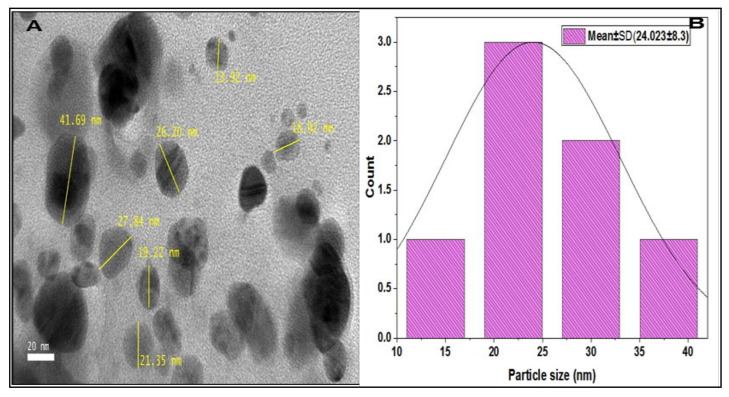
(**A**) HR-TEM micrograph of silver nanoparticles showing scattered, monodisperse, and spherical shape nanoparticles. (**B**) Histogram of average particles size of silver nanoparticles (×100,000).

The particle size, shape, and distribution of synthesized silver nanoparticles were analyzed using a high-resolution transmission electron microscope (HR-TEM). The HR-TEM produces clearer images with higher quality and resolution, about 3–6 times better than the conventional TEM. The results of the HR-TEM analysis confirm that nanoparticles synthesized are monodispersed with moderate size variation and spherical shape, with average particles sizes of 24.023 ± 8.3 nm. A few of them have an elliptical shape and are aggregated (Figure 4A,B).

### 2.7. Field Emission Scanning Electron Microscope (FESEM)

**Figure 5:**
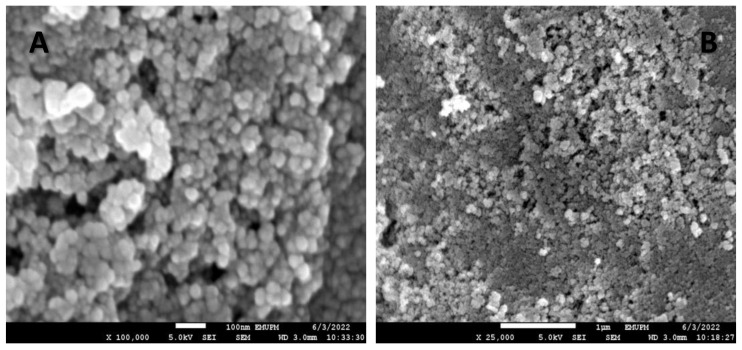
FESEM micrographs showing smooth surfaces and spherically shaped silver nanoparticles at magnifications of × 100,000 and × 25,000.

A field emission scanning electron microscope (FESEM) was used to analyze the topography of the surface area of synthesized silver nanoparticles because it produces an image with higher quality and resolution compared to a conventional scanning electron microscope (SEM). The FESEM micrographs showed that the nanoparticles synthesized have a smooth surface area and are spherical in shape without any significant agglomeration at different magnifications of × 100,000 and × 25,000 (Figure 5A,B). 

### 2.8. Energy Dispersive X-ray Spectroscopy (EDX)

**Figure 6:**
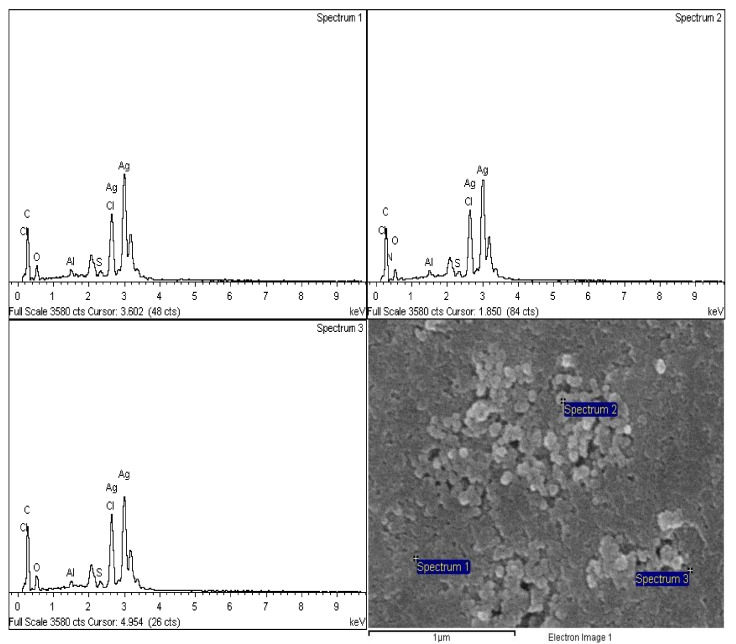
EDX spectra of synthesized AgNPs and FESEM micrograph showing EDX analysis regions.

The energy dispersive X-ray spectroscopy of the synthesized silver nanoparticles was conducted using FESEM equipped with an EDX detector. The EDX spectra of three regions of the FESEM micrographs were recorded (see Figure 6). The results of the EDX spectrum showed the existence of silver as the most common and abundant element contained in the sample, which showed a sharp peak at 3.0 keV in all the three regions selected (Figure 6). The percentages of elemental silver in all three regions are 40.42%, 40.44%, and 47.74%, respectively. Other elements such as carbon (36.11%, 33.54%, 29.34%), oxygen (15.04%, 11.37%, 14.34%), aluminium (0.47%, 0.48%, 0.39%), sulphur (0.50%, 0.57%, 0.58%), and chlorine (7.46%, 7.67%, 7.61%) were also recorded at low concentrations (Table 3).

### 2.9. Fourier Transform Infrared Spectroscopy (FTIR)

**Figure 7:**
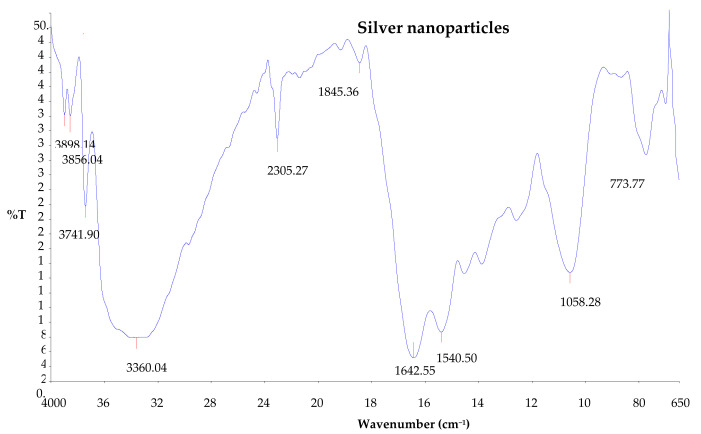
Fourier transform infrared spectrum of silver nanoparticles synthesized using *Citrus aurantifolia* peel extract.

The Fourier transform infrared (FTIR) analysis of the synthesized silver nanoparticles was conducted in order to identify the major functional groups or biomolecules on the surface of the synthesized silver nanoparticles that were possibly responsible for the synthesis and stabilization of the nanoparticles. The FTIR spectra of the synthesized silver nanoparticles showed intense peaks at 3898.14, 3856.04, 3741.90, 3360.04, 2305.27, 1845.36, 1642.55, 1540.50, 1058.28, and 773.77 cm^−1^ (Figure 7). The absorption bands appearing at 3898.14, 3856.04, 3741.90, and 3360.04 cm^−1^ show O-H stretching vibration of the hydroxyl group, and this indicates the presence of phenols and alcohols [1,27]. The peak at 1642.55 cm^−1^ shows C=C stretching vibration for alkene and C=N amine stretching [28,29]. Moreover, the band observed at 1540.50 cm^−1^ is due to the N-H bend of amide.

### 2.10. X-ray Diffraction Analysis (XRD)

**Figure 8:**
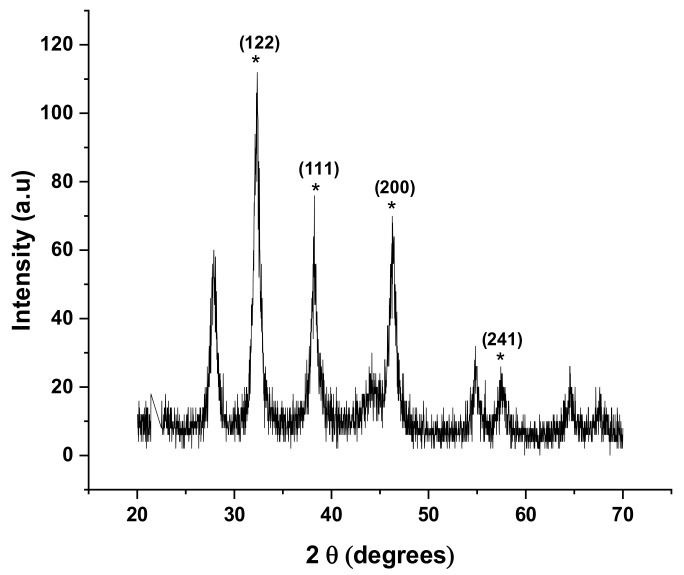
XRD Pattern of silver nanoparticles using CAFP extract.

The X-ray diffraction analysis was used to confirm the crystalline structure of the synthesized silver nanoparticles. The results of the analysis found that the synthesized silver nanoparticles using *Citrus aurantifolia* fruit peel extract showed diffraction peaks at 2θ values of 32.36°, 38.24°, 46.26°, and 57.44°, respectively. These 2θ values obtained were compared with the standard, and it was found that the obtained XRD spectrum peaks can be assigned to planes of (122), (111), (200), and (241) (Figure 8). The XRD pattern further clearly demonstrates that the synthesized silver nanoparticles are crystalline in nature. Moreover, the sharpening of the peaks clearly specifies that the particles are in nano-regime. These characteristics were compared to the standard powder diffraction card of the Joint Committee on Powder Diffraction Standards (JCPD No. 84-0713).

### 2.11. Dynamic Light Scattering (DLS) Analysis of Silver Nanoparticles 

**Figure 9:**
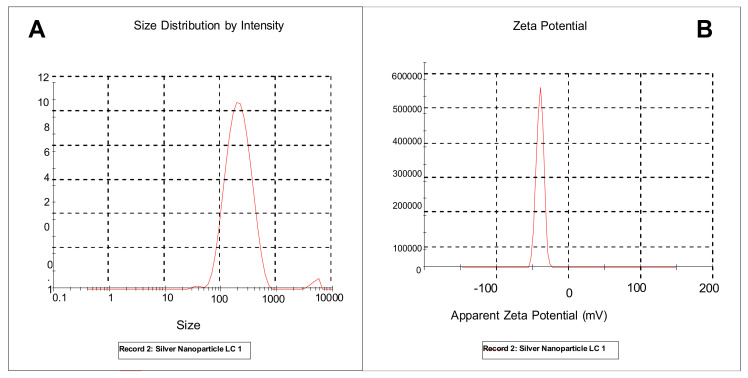
(**A**) Particle size distribution by the intensity of synthesized silver nanoparticles. (**B**) The zeta potential of the synthesized silver nanoparticles.

Dynamic light scattering (DLS) analysis determines the size distribution of particles, polydispersity index, and zeta potentials by measuring dynamic fluctuation of light scattering intensity caused by the Brownian motion of the particles. The results obtained showed the zeta potential value of −39.9 mV, and the obtained single peak indicated that the quality of the obtained silver nanoparticles is good and stable (Figure 9B). The average size of the silver nanoparticles synthesized was found to be 186.0 nm and with a Pdi value of 0.230 as measured by the DLS technique (Figure 9A).

## 3. Discussion

Plant secondary metabolites are generally classified into different classes according to their chemical structures; namely, flavonoids, tannins, phenols, alkaloids, steroids, saponins, glycosides, anthraquinones, etc. They are known to offer several health benefits to humans, such as antioxidant, antimicrobial, anti-inflammatory, and anti-allergic benefits [30,31]. For example, phenolic compounds are compounds with at least one hydroxyl aromatic ring substituted in their chemical structure, and this compound is known to have antioxidant activities [31,32]. Most importantly, these plant secondary metabolites have been reported to play an important role in the synthesis of silver nanoparticles as reducing, stabilizing, and capping agents [33]. 

In the present study, different groups of plant phytochemicals such as alkaloids, flavonoids, saponins, phenols, steroids, terpenoids, and tannins were found in *Citrus aurantifolia* fruit peel extract (CAFPE) through the qualitative phytochemical tests conducted. The presence of these chemical compounds in the plant’s extract gives insight into not only the ability of these chemical compounds to participate in the bio-reduction of silver ions (Ag^+^) but also the medicinal importance of *Citrus aurantifolia* peel extracts. This finding is similar to previously reported findings that also detected the presence of these chemicals in the peel extract of the *Citrus aurantifolia* plant [34]. Furthermore, the presence of these secondary metabolites in the plant extract in this study revealed the importance of phytochemicals in the synthesis of silver nanoparticles; for example, the synergistic interaction between these metabolites and silver ions that brings about the reduction of silver nitrate and the synthesis of silver nanoparticles. The negatively charged functional groups of plant metabolites present in the plant extract, such as carboxylate (COO^−^), and polar groups such as OH and C=O, have a high tendency to attach on the surface of the silver ion (Ag^+^), so these groups contribute to both the reduction and the stabilization of silver ions [1,33]. Moreover, phenolic compounds serve as antioxidants so that they can act as reducing agents in neutralizing free radicals and reducing metal ions [35]. Tannins, terpenoids, amides, and ketones can also donate electrons to reduce the silver ions (Ag^+^) [36]. Therefore, the presence of secondary metabolites such as flavonoids, tannins, and saponins in the peel extract of the *Citrus aurantifolia* fruit in this study was strongly suspected to play a role in the biosynthesis of silver nanoparticles. 

The formation and synthesis of silver nanoparticles using the peel extract of *Citrus aurantifolia* in the present study were confirmed by the color change of the reaction mixture of silver nitrate solution and the plant extract from a slightly yellow color to dark brown after 30 min of the reaction. The change in color indicates the formation of colloidal silver nanoparticles. However, the possible explanation for the color change of the solution from yellow to dark brown could be as a result of a change in the plasmon resonance of silver nitrate because of the reduction process [37]. This suggests that there is a formation of elemental silver with nano dimensions from the ions in the silver nitrate solution. The basis of synthesis and formation of silver nanoparticles can be further explained in two different steps; in the initial stage, silver atoms are formed due to the reduction of various complexes with silver ions (Ag^+^), followed by the second step in which there is a formation of oligomeric clusters due to agglomeration, and these clusters eventually lead to the formation of colloidal silver nanoparticles [38]. Moreover, the UV-vis absorption spectra of the synthesized silver nanoparticles in the present study revealed an absorption peak that shifted from 360 to 375 nm, 375 to 385 nm, and 385 to 405 nm, as the reaction time increased from 30 min, 1 h, 1 h and 30 min, and 2 h. The surface plasmon resonance peak appeared at about 360 nm at the first 30 min of the reaction and slowly shifts to the longer wavelength with increasing reaction time, which indicates the increase in quantity or size of the silver nanoparticles [20]. The increase in surface plasmon resonance intensity peak might be due to the transformation of the hydroxyl (OH) functional groups to carbonyl groups (C-O) because of the silver ions’ reduction [39].

Several applications of biologically synthesized silver nanoparticles are dependent on various parameters, such as the sizes, shapes, and crystallographic structures of the nanoparticles. For instance, the biological activities of nanoparticles such as larvicidal, antimicrobial, and cytotoxic effects depend on the nanoparticle size; the smaller the nanoparticle size, the greater the activity compared to larger nanoparticles [40]. The HR-TEM and FESEM micrographs of the synthesized silver nanoparticles in the current study confirmed that the nanoparticles are spherical in shape, monodisperse, and have an average particle size of 24.02 nm, respectively. Similar findings have been reported for silver nanoparticles with a size range of 10–25 nm, synthesized using plant extracts as reducing agents [6].

The hydrodynamic size, zeta potential, and polydispersity index of synthesized nanoparticles in a suspension were determined using dynamic light scattering (DLS) in the current study. In dynamic light scattering, the detection of the light scattered from the interaction of light with matter or a sample provides information related to the physical characteristics of the sample. Mostly, in dynamic light scattering experiments, a monochromatic beam is directed to the sample and then a detector records the certain light at a certain angle [41]. The average particle size of the silver nanoparticles synthesized in dynamic light scattering was 186 nm. This finding is contrary to what was obtained in the HR-TEM results for 24.02 nm and 25.7 nm. The possible explanation for this finding is that DLS gives the size of the nanoparticle plus the liquid layer around the particle, whereas size measured by HR-TEM gives the actual size of the nanoparticle [42]. The polydispersity index (PDI) is a measure of homogeneity or heterogeneity of a sample based on size, and it is also measured using dynamic light scattering. The PDI value of the *Citrus aurantifolia* synthesized silver nanoparticles was 0.23, which indicates that the synthesized silver nanoparticles are monodisperse or homogeneous. Generally, PDI is measured between 0 and 1 [42]. We also obtained the zeta potential value of −39 mV, which indicates that the synthesized silver nanoparticles have good stability. The electrophoretic mobility and charge of each particle are measured using zeta potentials; when the zeta potential value is high, it indicates a high electric charge on the surface of the nanoparticles, describing a strong repulsive force among the particles that prevents aggregation and leads to nanoparticle stabilization [42].

In the current study, the XRD patterns of the synthesized nanoparticles indicate that the nanoparticles are crystalline in nature. The shape peaks seen in the pattern clearly specify that the particles are in nano-regime. The elemental composition of the *Citrus aurantifolia* fruit peel (CAFP) synthesized silver nanoparticles was analyzed using energy dispersive X-ray spectroscopy (EDX), and the results show that silver had the highest peak, which clearly indicates that silver is the major element presence. However, the presence of other elements such as carbon, oxygen, aluminium sulphur, and chlorine was also recorded at low concentrations. The presence of carbon and the other elements may be the result of the carbon adhesive tape used in coating the sample during the preparation, residual phytoconstituents in the plant extract used in the synthesis, or contaminants introduced into the sample during the preparation. This finding is in line with previously reported findings in the literature [7].

## 4. Materials and Methods

### 4.1. Plant Material and Chemicals

The dried peel powder of *Citrus aurantifolia* fruit was purchased from the Chemical Engineering Pilot Plant, Universiti Teknologi Malaysia (UTM), Johor Bahru, Malaysia. For the synthesis of silver nanoparticles and phytochemical screening of the plant extract, Dragendorff‘s reagents, 10% ferric chloride (FeCl_3_), sodium hydroxide (NaOH), sulphuric acid (H_2_SO_4_), silver nitrate (AgNO_3_), 10% ammonia solution, methanol, petroleum ether, and chloroform were purchased from a certified supplier (Biotek Abadi Sdn Bhd, Malaysia). All the chemicals used were of analytical grade and were used without any purification.

### 4.2. Plant Extraction

The plant extraction was conducted using two different solvents that include distilled water and methanol, respectively. One hundred grams (100 g) of the peel powder of *Citrus aurantifolia* fruit was weighed using a digital weighing scale (OEM DT580, Jiangsu, China) and macerated in 500 mL of solvents at the ratio of 1:5 *w/v*. The solutions were maintained by constant shaking at 167 rpm for 24 h using a hybridization incubator shaker machine (Combi-SV2DX, Seoul, Republic of Korea). The crude extract was then filtered using a muslin cloth and later filtered using Whatman No. 1 filter paper. The aqueous extract was then kept in a refrigerator at 4 °C until further use, whereas the methanolic extract was concentrated using a rotary evaporator at 40 °C [43]. The methanolic extract was used for the identification of the chemical compounds present in the plant extract using gas chromatography mass spectrometry (GC–MS), whereas the aqueous extract was used for the synthesis of the silver nanoparticles. 

### 4.3. Preliminary Phytochemical Analysis 

The preliminary phytochemical screening (qualitative) of the plant extract was carried out to determine the presence of different bioactive compounds in the plant extract (flavonoids, alkaloids, tannins, saponins, phenols, terpenoids, steroids, anthraquinones, and glycoside). Results of the qualitative phytochemical analysis were expressed as (+) positive and (−) negative. The phytochemical analysis was conducted as described previously by Ezeonu and Ejikeme (2016) [44] with some modifications.

#### 4.3.1. Test for Alkaloids (Dragendorff’s Test)

One milliliter (1 mL) of Dragendorff’s reagents was added to a test tube containing filtrate. The formation of an orange red precipitate shows the presence of alkaloids [45]. 

#### 4.3.2. Test for Saponins (Honeycomb Test)

Four milliliters (4 mL) of the aqueous plant extract solution were added to a test tube containing 10 mL of distilled water. The test tube containing the mixture was agitated vigorously for 5 min, and allowed to stand for 25 min and observed for honeycomb froth, which indicated the presence of saponins [46].

#### 4.3.3. Test for Tannins (Ferric Chloride Test)

A few drops of a 10% ferric chloride solution were added to 2 mL of aqueous plant extract. The occurrence of blackish blue or green blackish coloration indicates the presence of tannins [32]. 

#### 4.3.4. Test for Flavonoids (Shibita’s Test) 

Five milliliters (5 mL) of sodium hydroxide (NaOH) were added to 5 mL of the plant extract in a test tube. The formation of a yellow color indicates the presence of flavonoids in the plant extract [32].

#### 4.3.5. Test for Phenols (Ferric Chloride Test)

Two milliliters (2 mL) of the plant extract were added to 2 mL of distilled water. Thereafter, 10% of ferric chloride solution was added to the mixture. The presence of phenols is indicated by the presence of a bluish black color [30].

#### 4.3.6. Test for Terpenoids (Sulphuric Test)

The plant extract was dissolved in 3 mL of chloroform. This was then evaporated to dryness, and 2 mL of concentrated sulphuric acid (H_2_SO_4_) was added and heated for about 3 min. A reddish brown color indicated the presence of terpenoids [30,47].

#### 4.3.7. Test for Steroids (Test for Salkowski) 

Five milliliters of the plant extract were added to 2 mL of chloroform. Thereafter 2 mL of sulphuric acid (H_2_SO_4_) was carefully added to the mixture to form a lower layer. The mixture was then observed for a reddish brown color which indicates the presence of steroids [34].

#### 4.3.8. Test for Anthraquinones (Borntrager’s Test)

Two grams (2 g) of plant extract powder was weighed and dissolved in 20 mL petroleum ether/chloroform and later filtered using a filter paper. An equal volume of aqueous ammonia solution was added to the filtrate. The solution was shaken, and the upper aqueous layer was observed for bright pink coloration as indicative of the presence of anthraquinones [43].

### 4.4. Gas Chromatography–Mass Spectrometry (GC–MS) Analysis 

The gas chromatography–mass spectrometry (GC–MS) analysis of the methanolic plant extract of *Citrus aurantifolia* fruit peel was carried out using GC–MS (QP 2010 series, Shimadzu, Tokyo, Japan). For GC–MS analysis of the plant extract, an electron ionization system with ionization energy of 70 eV was used. Helium gas is used as a carrier gas at a constant flow rate of 1.51 mL/min. The injector and mass transfer line were set at a temperature of 200–240 °C. The temperature of the oven was also set between 70 and 220 °C at 10 °C/min. About 50 μL prepared sample with methanol (HPLC grade) was introduced into the injector in the split-less mode with a split ratio of 1:40 and with mass scan of 50–600 amu. The GC–MS analysis running time was set at 35 min. The relative percentage of the plant extract constituents was presented as a percentage with peak area normalization. Percentage of the extract constituents was expressed as a percentage with peak area normalization. The identification of the chemical compounds was conducted by comparing the spectrum of unknown chemical compounds with the known spectrum of the chemical compounds in the NIST structural library to identify the names, molecular weight, molecular formula, and structure.

### 4.5. Fourier Transform Infrared Spectroscopy (FTIR) of Plant Extract

The FTIR analysis of the plant extract was carried out using the aqueous extract of *Citrus aurantifolia* fruit peel. A small drop of the aqueous plant extract was placed on one of the KBr plates using a micropipette; the second plate was placed on top and made a quarter turn to form a thin and transparent KBr pellet. The pellet-containing plate was placed on the sample holder and run through a spectrum. The FTIR measurements of the absorption bands were observed in the regions of 650 to 4000 cm^−1^ at 4 cm^−1^ resolution. The fingerprints of the chemical compounds generated were presented as in the FTIR spectra [48,49]. 

### 4.6. Silver Nanoparticles (AgNPs) Synthesis Using CAFP Extract

The synthesis of silver nanoparticles (AgNPs) was conducted following the method previously described by Nur Syazana et al., (2018) [29], with some modifications. An aqueous solution of one milli molar (1 mM) of silver nitrate was prepared using distilled water and silver nitrate compound (AgNO_3_). Thereafter, 50 mL of plant extract was added to 450 mL solution of 1 mM of silver nitrate (AgNO_3_) at a ratio of 1:9. The mixture was then kept in a water bath at 70 °C and allowed to incubate for 2 h. During the 2 h, the reduction of silver ions was monitored by the UV-visible spectra of the reaction mixture at time intervals of 30 min, 1 h, 1 h and 30 min, and 2 h using a UV-Visible spectrophotometer. The formation of silver nanoparticles was determined by the color change of the mixture from yellow to brown because of surface plasmon resonance. The mixture containing the colloidal nanoparticles was centrifuged at 4000 rpm for 20 min using a centrifuge machine (Hettich Rotofix 32 A, Buford, MA, USA) and the pellets obtained were lyophilized. The synthesized silver nanoparticles were stored until further analysis [7]. 

### 4.7. Characterization of Silver Nanoparticles (AgNPs)

#### 4.7.1. Visual Observation 

The mixture of the plant extract and silver nitrate (AgNO_3_) suspension was observed for a color change to brown color, which indicates the formation of silver nanoparticles (AgNPs). The color change in the suspension has been reported to be a result of surface plasmon vibrations of the silver nanoparticles [50]. 

#### 4.7.2. UV-Visible Spectrophotometry Analysis

The optical properties of the synthesized silver nanoparticles (AgNPs) were determined using a UV-Visible spectrophotometer (Beckman Coulter DU730, South San Francisco, CA, USA). About 3 mL of the sample was analyzed in the wavelength range of 200 nm to 800 nm at room temperature. The results of the absorption spectra were presented in graph of absorbance as a function of wavelength (nm) [12].

#### 4.7.3. High Resolution Transmission Electron Microscope (HR-TEM)

The size and morphology of the synthesized silver nanoparticles (AgNPs) were analyzed using a high-resolution transmission electron microscope (HR-TEM). The morphological analysis of the synthesized silver nanoparticles (AgNPs) was carried out following the methods described previously by Ahmad and Sharma (2012) [51], with a few modifications. A dried powder of silver nanoparticles was prepared by dispersing a desired amount of the powder in acetone and ultrasonicated for 5 min. The sample was placed on a copper grid coated with carbon film and dried at room temperature for 15 min. The HR-TEM measurements were performed on a Technai G2 F20S-TWIN (FEI, Hillsboro, OR, USA). Average diameters and shapes of silver nanoparticles were determined from the HR-TEM micrographs, while the distribution of the nanoparticles was determined by counting the approximate number of nanoparticles on the micrograph. The photomicrograph obtained was analyzed using Image J (version 1.5 NIH) and Origin Lab software (student version 2022). The results of the average size and distribution of the nanoparticles were presented in a histogram. 

#### 4.7.4. Field Emission Scanning Electron Microscope (FESEM) 

The surface morphology of the synthesized silver nanoparticles was analyzed using a field emission scanning electron microscope (FESEM). A dried powder of silver nanoparticles was used for the FESEM analysis. The FESEM analysis was conducted by coating the synthesized silver nanoparticles with a fine layer of platinum and placing them in the FESEM to view their surface image. The micrographs of the surface morphology of silver nanoparticles were recorded using a field emission scanning electron microscope. 

#### 4.7.5. Energy Dispersive X-ray Spectroscopy (EDX)

A field emission scanning electron microscope (FESEM) equipped with energy dispersive X-ray spectroscopy was used for the EDX analysis of the samples. The EDX analysis was conducted in order to determine the elemental composition and abundance of the biosynthesized silver nanoparticles. A small amount of the silver nanoparticles was placed on a carbon-coated grid and transferred to the microscope. After studying the micrograph of the silver nanoparticles, three regions from the micrograph were selected and analyzed for the EDX analysis, and spectra were recorded. 

#### 4.7.6. Fourier Transform Infrared Spectroscopy (FTIR) Analysis

The Fourier transform infrared spectroscopy (FTIR) of the synthesized silver nanoparticles was carried out according to method previously described by Awwad et al., (2013) and Erdogan et al., 2019 [52,53] with some modifications. The analysis was conducted using Thermo 6700 (Thermo Fisher Scientific, Waltham, MA, USA) to determine the functional groups that led to the formation of silver nanoparticles. The sample was placed into the FTIR sample holder and analyzed. The FTIR measurements of the absorption bands were recorded in the regions of 650 to 4000 cm^−1^ at 4 cm^−1^ resolution, and the fingerprints of chemical compounds generated were identified by FTIR analysis. 

#### 4.7.7. X-ray Diffraction Analysis (XRD)

The X-ray diffraction analysis of the synthesized silver nanoparticle powder was conducted to determine the crystalline structural pattern of the nanoparticle. Silver nanoparticle powder was mounted on the central plane of the X-ray diffractometer (XRD-6000, Shimadzu, Japan) and studied using Cu-kα radiation source in the range of 20°–70° with a 2 °/min scan speed in continuous scan mode. The generator voltage and current were set at 30.0 kA and 30.0 mA, respectively. 

#### 4.7.8. Dynamic Light Scattering (DLS) and Particle Size

The average particle size, distribution, and zeta potential of the synthesized silver nanoparticles were determined using zetasizer (Nano ZS-90, Malvern Instruments, Malvern, UK). The analysis was conducted following the method previously described by Erdogan et al. (2019) [50], with some modifications. An aliquot of the synthesized silver nanoparticles powder was sonicated for 15 min in 15 mL of deionized water. Three milliliters of the suspension were then filtered through a 0.20 μm pore-sized syringe, and then the suspension was analyzed. The average particle size distribution and zeta potential were determined by measuring the dynamic fluctuation of the light intensity of the colloidal particles. The measurement of the average hydrodynamic diameter of the particles, peak values in the hydrodynamic diameter distribution, and polydispersity index (PDI) were recorded and analyzed using zeta sizer software (version 7.11). All measurements were carried out in triplicate with a temperature equilibration time of 1 min at 25 °C. The results were presented in a graph showing the peaks and values. 

## 5. Conclusions

In the current study, silver nanoparticles were successfully fabricated via the reduction of silver ions (Ag^+^) by the secondary metabolites present in *Citrus aurantifolia* fruit peel extract. Different types of secondary metabolites such as flavonoids, alkaloids, tannins, saponins, phenols, and steroid were identified in the plant extract. Hence, *Citrus aurantifolia* fruit peel extract can act as a good candidate for the reduction of silver ions to silver nanoparticles. It is concluded that the green synthesis approach appears to be a cost-effective alternative to the conventional physical and chemical methods. Moreover, the CAFPE can be potentially used for the large production of silver nanoparticles for various applications. The present study contributes to the understanding of green synthesis of silver nanoparticles using plant extracts. 

## Figures and Tables

**Table 1:** Phytochemical compounds present in the *Citrus aurantifolia* plant extract.

Plant Species	Phytochemicals	Status
*Citrus aurantifolia*	Flavonoids	+
	Alkaloids	+
	Tannins	+
	Saponins	+
	Phenols	+
	Terpenoids	+
	Steroids	+
	Anthraquinones	−

**Table 2:** Chemical compounds of peel extract of *Citrus aurantifolia* fruit and their abundance.

Peak	RT Time (min)	Compound Names	Peak Area %	MW (Da)
67	17.630	Tris (tert-butyldimethylsilyloxy) arsane	2.017	342.4
46	15.343	Cyclotrisiloxane, hexamethyl-	2.10	468.7
39	10.584	7-Pentadecyne	2.16	210.4
16	10.237	2-Pyridinamine, N-(4,5-dihydro-5-m ethyl-2-thiazolyl)-3-methy	2.68	94.11
13	11.526	Eicosane, 2-methyl-	2.63	408.4
27	14.704	4-Methyl-2-trimethylsilyloxy-acetophenome	3.55	222.3
40	14.203	1,2,4-Benzenetricarboxylic acid	4.42	210.1
38	13.691	1,1,1,3,5,5,5-Heptamethyltrisiloxane	6.48	468.7
35	9.449	Fumaric acid, nonyl pentadecyl	2.81	116.0
10	13.234	Cyclotrisiloxane, hexamethyl	8.16	282.5

**Table 3:** The percentage of elemental composition of silver nanoparticles.

Spectrum	C	N	O	Al	S	Cl	Ag	Total %
Spectrum 1	36.11		15.04	0.47	0.50	7.46	40.42	100.00
Spectrum 2	33.54	5.94	11.37	0.48	0.57	7.67	40.44	100.00
Spectrum 3	29.34		14.34	0.39	0.58	7.61	47.74	100.00
Maximum	36.11	5.94	15.04	0.48	0.58	7.67	47.74	
Minimum	29.34	5.94	11.37	0.39	0.50	7.46	40.42	

## Data Availability

Not applicable.
